# Supplementary material for: A Modular Microscale Granuloma Model for Immune-Microenvironment Signaling Studies in vitro
Source: Front Bioeng Biotechnol. 2020 Aug 18;8:931. doi: 10.3389/fbioe.2020.00931 (PMC7461927; doi:10.3389/fbioe.2020.00931)
Supplement: Supplementary file 1 [file Presentation_1.pdf]

A modular microscale granuloma model for immune-microenvironment signaling studies *in vitro*

Samuel B. Berry[1], Maia S. Gower[1], Xiaojing Su[1], Chetan Seshadri[2], Ashleigh B. Theberge[1,3*]
[1]Department of Chemistry, University of Washington, Seattle, WA USA
[2]Department of Medicine, University of Washington, Seattle, WA USA
[3]Department of Urology, University of Washington, Seattle, WA USA
[*]Corresponding author: Dr. Ashleigh Theberge, abt1@uw.edu

The supporting information for "A modular microscale granuloma model for immune-microenvironment signaling studies *in vitro*" includes information that readers might find useful for adapting this platform for their own research and laboratory setups. We include detailed technical schematics of our Stacks platform, the code for the ImageJ macro we adapted for analysis of endothelial morphology, device preparation protocols, experimental optimization considerations, and design files for the devices used in this manuscript.

A. 3D Injection Molded Device for Model Granuloma Layer

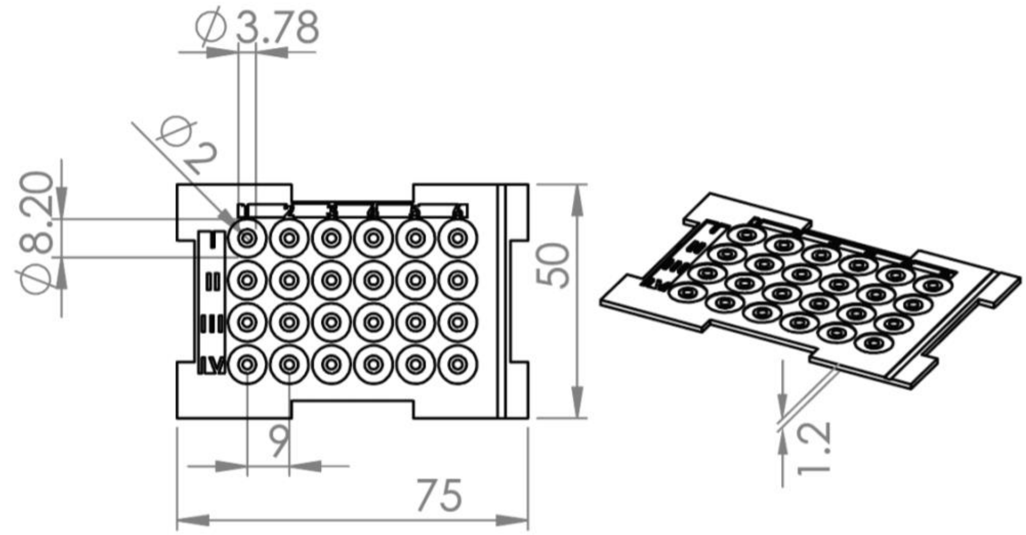

B. CNC-Milled Device for Endothelial Layer

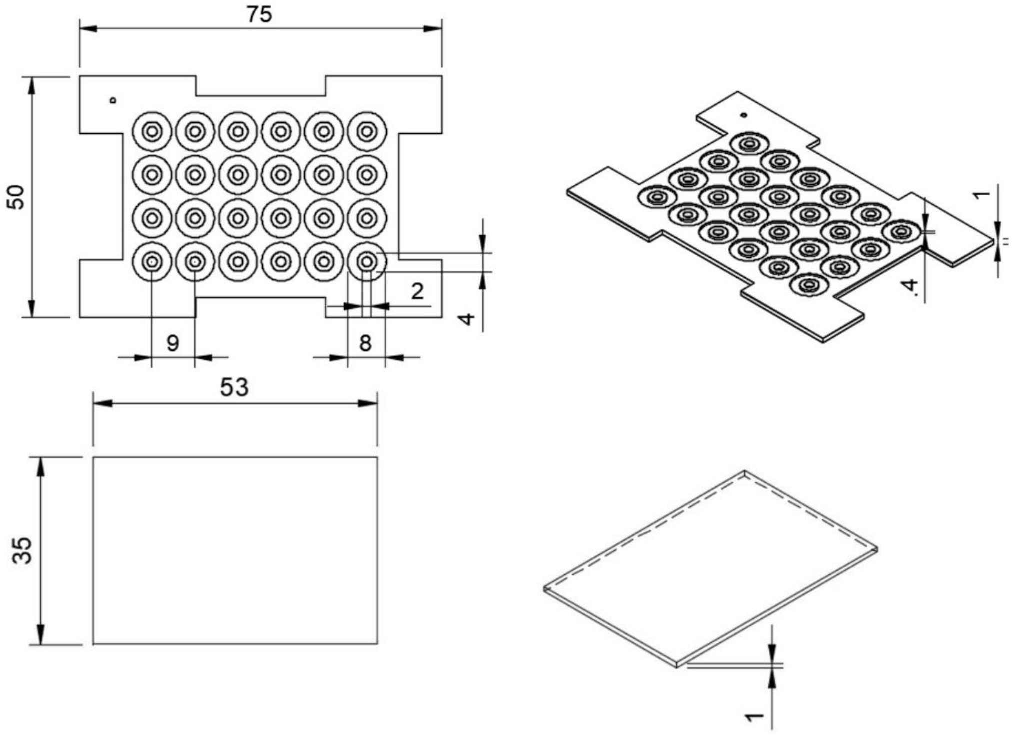

<u>Supplemental Figure 1:</u> Detailed Device Schematics and Dimensions. A) The injection molded layer used in Figures 1-4 for the model granuloma. Dimensions and figure reproduced from Yu et al.[1], Supplemental Figure 3. B) The endothelial layers containing a floor used in Figure 4 was milled using CNC bonding with a solvent-bonded floor. Dimensions labeled in millimeters.

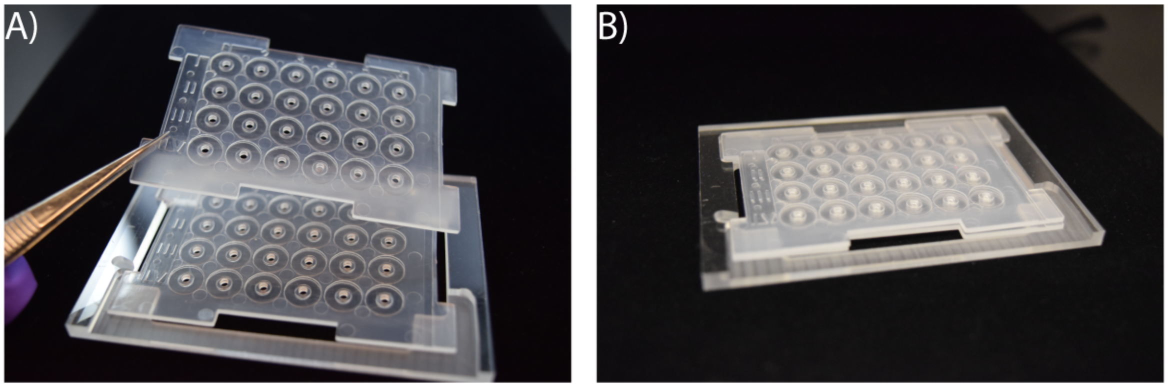

<u>Supplemental Figure 2:</u> Stacks devices placed inside of CNC-milled device holders. A-B) Layers can be placed directly into the holders to prevent contact between the open suspended culture wells and the floor, as well as to keep the devices in place during culture and aligned once vertically stacked.

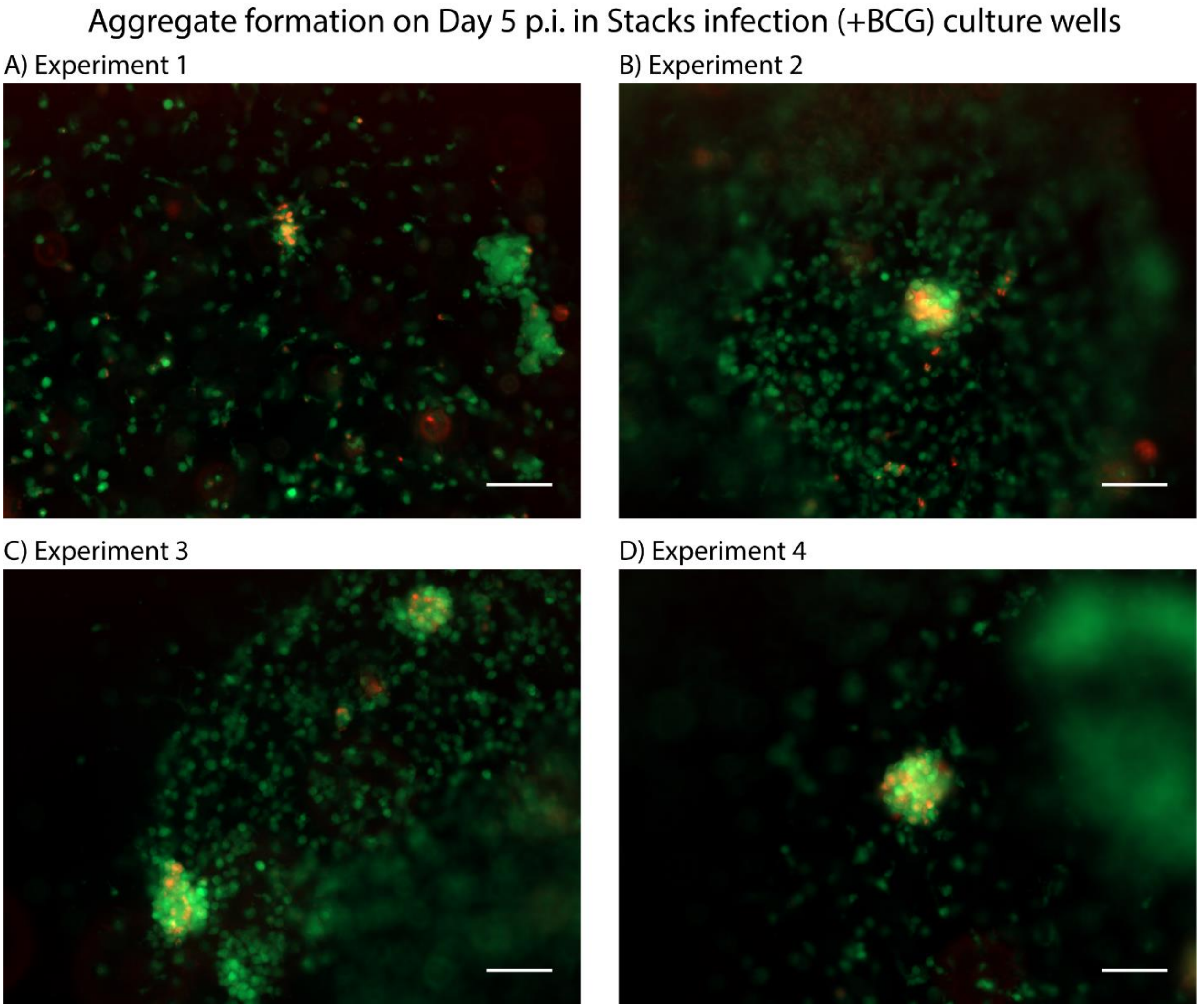

Supplemental Figure 3: Representative images of aggregate formation on Day 5 p.i. in each independent experiment demonstrate retention of the multicellular aggregates within the Stacks infection (+BCG) culture wells. Monocyte-derived macrophages (MDMs) (CellTracker Green) and *M. bovis* BCG (mCherry) form these aggregates within a 3D collagen plug. Images were obtained using standard fluorescence microscopy. CellTracker Green staining of the MDMs enabled visualization of these aggregates with live cells (no fixing), as CellTracker Green stains live cells and doesn't require additional staining steps. Scale bars: 100 µm.

<u>Supplementary Table 1: Timeline of Aggregate Formation in Stacks Platform</u>

Extent of aggregate formation observed in Stacks infection culture wells					
Replicate Number	*Day 1 p.i.*	*Day 2 p.i.*	*Day 3 p.i.*	*Day 4 p.i.*	*Day 5 p.i.*
Experiment 1	1	N/A	N/A	3	3
Experiment 2	1	2	2	3	3
Experiment 3	1	2	2	3	3
Experiment 4	1	2	2	3	3

1: no aggregate formation observed in Stacks infection culture wells
2: aggregate formation observed in minority of wells/progressive formation of aggregates
3: aggregate formation observed in majority of wells in device
N/A: no observations noted in lab notebook
p.i.: post infection

At Day 1 p.i., no aggregates were observed within the infected (+BCG) culture wells within the Stacks device. Starting Day 2 p.i., aggregate formation was observed in a minority of the wells, with progressively more aggregates forming on Day 3 p.i. By Day 4 p.i., aggregates were observed in the majority of wells. Due to the microscale volume of media used to feed each well (8 μL) and the open characteristics of the platform, changes in humidity and temperature can have significant effects on evaporation and culture temperature; therefore, wells were not imaged daily so as to minimize the amount of time the cultures were out of the humidified cell culture incubator; qualitative observations are provided here to illustrate an overview of the aggregate formation timeline for future users.

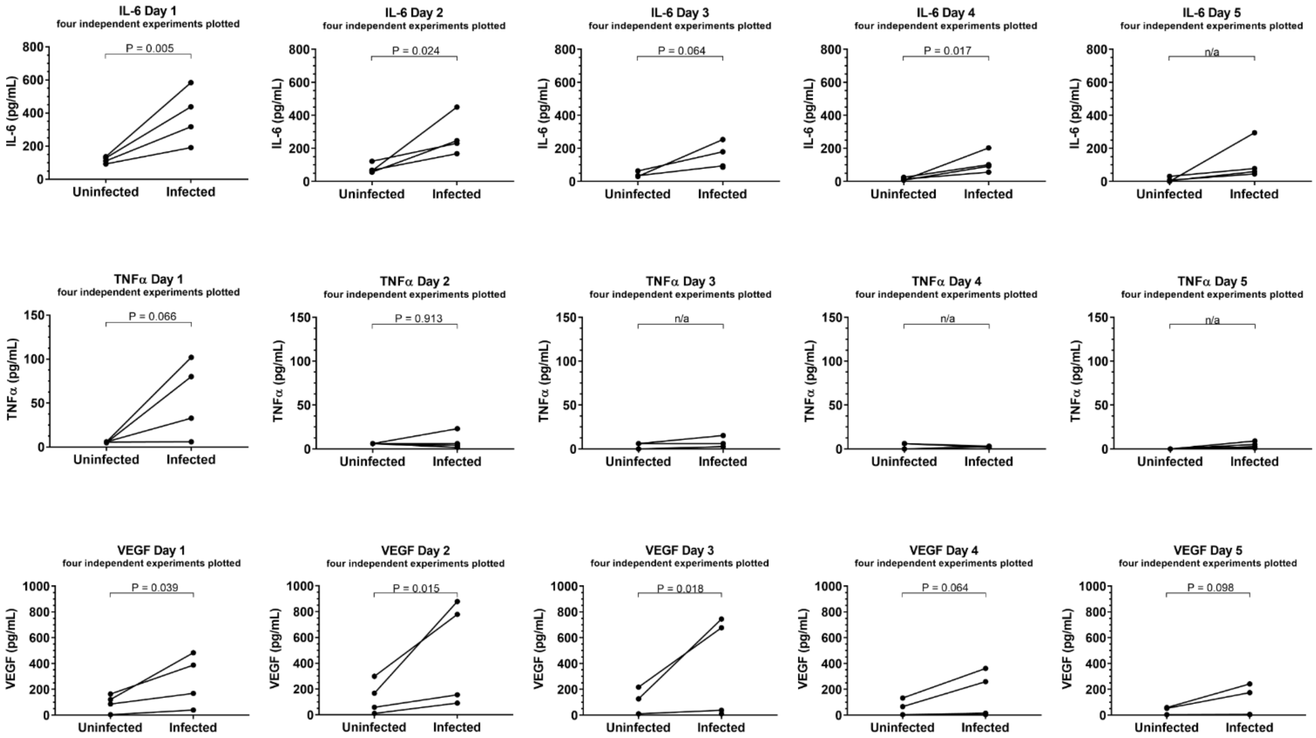

<u>Supplemental Figure 4</u>: Model granuloma layers secrete proinflammatory cytokines following infection (Day 1 p.i.). Individual cytokine profiles from each independent experiment across all days are included to demonstrate the model's signaling ability over time across replicates. $P < 0.05$; ratio paired *t*-test; "n/a" indicates that the ratio paired *t*-test could not be run as the sample contained values equal to zero (signal equal to or below blank standards).These data are the expanded results from the average data shown in Figure 3.

Supplementary Information 1: Polypropylene (PP) Device Flattening Protocol

Protocol is for use with a Carver Bench Top Standard Heated Press (Model #4386). This protocol can be adapted for use with alternative heated presses with model-specific changes.

1. Preheat platens to 110°C (this takes ≈ 20 min to heat up and stabilize, as the temperature fluctuates).
2. Prepare a stack of devices (4-5 devices) and carefully align the stack to avoid deformation.
3. Place Kapton® polyimide film (#2271K2, McMaster Carr) on the top and bottom platens and place the stack of devices between the films (to avoid direct contact between the devices and the platens).
4. Turn on the pressure sensor.
5. Pump the hot press handle until contact is just made and the devices are flattened.
   - The devices stacks should not be compressed at all (i.e., the pressure sensor should read "0 psi" or fluctuate between “0-10” psi), but the devices should be in contact with the platens and flattened.
6. Let the stacks flatten for 60 min at the above temperature and pressure.
7. After 60 min, turn off the platen temperature and let the temperature drop down to ≈30°C.
   - Do not use coolant to lower the temperature as this causes the temperature to drop too rapidly and can cause deformation. This cooling process takes 1.5-2 h.
8. Once the temperature has dropped below 30°C, turn on the coolant circulator and run the coolant for 5 min to completely cool the platens.
9. Turn off the coolant, release the pressure, and remove the devices from the platens.

Supplementary Information 2: Culture Optimization for Miniaturization of Mycobacterial Infection

Condition	Conventional Macroscale Protocol	Optimized Microscale Protocol	Reason for Optimization
Fetal Bovine Serum Concentration[2]	10%	15%	Adaptation for microculture leads to decreased volume of media per cell; increased [FBS] provides greater amount of serum to culture.
Media Buffer Concentration[2]	Variable (0-25 mM HEPES)	25 mM HEPES	Adaptation for microculture leads to decreased volume of media per cell; increased [HEPES] provides greater buffering capacity of media to counter waste accumulation.
Culture Feeding Frequency[2]	Every 2nd or 3rd day	Daily	Adaptation for microculture leads to decreased volume of media per cell; increased media changing frequency prevents nutrient depletion and waste buildup.
Multiplicity of Infection (MOI)	0.1[3,4]	0.05	Adaptation to microscale decreases the total volume of space in the culture well and increases probability of BCG-recognition by monocyte-derived macrophages; decreased MOI results in reproducible and consistent aggregate formation in each well with sufficient uninfected MDMs remaining to aggregate around the infected cells.
Tween-80 Concentration	0.05%	0.003%	Adaptation for the Stacks platform requires a Tween-80 concentration below the critical micelle concentration to maintain capillary pinning and functionality of Stacks devices (see below).
Device Material	Polystyrene (PS)	Polypropylene (PP)	Adaptation for use with immune cell and mycobacterial media requires an increase in the contact angle to maintain capillary pinning of media for cultures.

Optimization of the Tween-80 concentration for culture of *M. bovis* BCG was required for compatibility with the Stacks platform. Traditionally, mycobacteria are cultured in varying concentrations of surfactant (commonly Tween-80) to prevent clumping of mycobacteria during culture. However, the presence of surfactant within the Stacks platform interferes with the capillary pinning necessary for maintenance of cell cultures and stacking of multiple layers. This is due to the decrease in interfacial tension between the culture media and the device surface caused by the presence of surfactant, effectively decreasing the contact angle of the media on the surface. In order to alleviate the effects of surfactant on the capillary pinning, the concentration of surfactant must be well below the critical micelle concentration (CMC), which is 0.0013% w/v[5], in the final media. Therefore, we cultured the BCG at a concentration of 0.003% w/v Tween-80, which yields a final concentration (after all dilutions and mixing) of 0.0000025% w/v Tween-80 in the collagen plug; this concentration was selected as it was the highest concentration of Tween-80 we could use without loss of capillary pinning on the surface. However, to decrease the aggregation of BCG within our model system, the BCG were vortexed, vigorously pipetted, passed through a 27G needle to disperse aggregates, and then allowed to settle for ≈ 1 min before aliquoting from the top of the culture for use.

Supplementary Information 3: Culture Optimization of Endothelial Layer

To miniaturize the *in vitro* angiogenesis model[6-8] we adapted for this layer and to ensure functionality, we optimized certain components of the culture system within the Stacks platform. The first condition optimized was the seeding density; too high of a seeding density results in formation of a confluent monolayer or islands of cells on the surface of the Matrigel, whereas too low of a seeding density results in a dispersed culture that does not form endothelial connections. Therefore, the seeding density was calculated from previously established protocols using 48 well plates[7] to obtain a similar ratio of cells to area in the Stacks well (1,650 cells/well of the Stacks layer). We observed that concentrations greater or less than 1,650 cells/well resulted in inconsistent tubule or network formation.

Additionally, the volume of Matrigel within each well was optimized. The final volume of 3 μL was selected to allow complete and reproducible coating of the bottom of each well and to limit the effect of the meniscus on cell localization (i.e., cells aggregating towards the middle of the well/bottom of the meniscus) and cell visualization. Lower volumes of Matrigel (< 3 μL) resulted in uneven coating of the surface of the well floor, causing a non-uniform surface wherein cells adhered to both Matrigel and exposed polystyrene on the floor of the well, leading to altered morphology. Higher volumes of Matrigel (> 3 μL) caused an exaggerated meniscus effect, resulting in cell aggregation in the center of the well. In cases where the volume of the well was completely filled with Matrigel (> 3.6 μL), we observed cell growth both on the Matrigel and on the polystyrene pinnng ridge surrounding the well, resulting in varying surface-dependent morphologies (i.e., cells displayed different morphologies on the polystyrene and the Matrigel).

Supplementary Table 2: Files Included in Supplemental Information

Figure	File Name
Figure 1-4 – Injection Molded Device[1] (3D) Model Granuloma Layer	*3D Injection Molded Layer.f3d*
Figure 1-4 – CNC-Milled Device Holder	*Stacks Device Holder.f3d*
Figure 4 – CNC-Milled Device (2D) Endothelial Layer	*2D CNC Milled Layer.f3d*<br>*2D CNC Milled Floor.f3d*
Figure 4 – ImageJ Macro for Analysis	*ImageJ Macro Image Preparation and Analysis.txt*

The file for the injection molded device was reproduced from Yu et al.[1]

References:

1. Yu, J. et al. "Reconfigurable open microfluidics for studying the spatiotemporal dynamics of paracrine signaling". *Nat. Biomed. Eng.*, 2019, 3, 830-841.
2. Su, X. et al. "Effect of microculture on cell metabolism and biochemistry: do cells get stressed in microchannels?" *Anal. Chem.*, 2013, 85, 3, 1562-70.
3. Kapoor, N. et al. "Human granuloma *in vitro* model, for TB dormancy and resuscitation". *PLoS ONE*, 2013, 8, 1, e53657.
4. Puissegur, M.P. et al. "An *in vitro* dual model of mycobacterial granulomas to investigate the molecular interactions between mycobacteria and human host cells", *Cell. Microbio.*, 2004, 6, 5, 423-433.
5. Sigma-Aldrich, "Tween® 80 Sigma Ultra Product Information Sheet", P8074. Accessed 2020/04/01.
6. Koh, W. et al. "In vitro 3D collagen matrix models of endothelial lumen formation during vasculogenesis and angiogenesis". *Methods in Enzymology*, 2008, 443.
7. Lonza, Inc. "Clonetics™ Endothelial Cell System Technical Information and Instructions*;"* Walkersville, MD. 2018.
8. Sarkanen et al. "Intra-laboratory pre-validation of a human cell based in vitro angiogenesis assay for testing angiogenesis modulators". *Front. Pharmacol*. 2010; 1:147.
